# Resolution of occult anastomotic stricture with anal dilator: challenges with the conventional diagnostic criteria in low anterior rectal resection patient—a case report

**DOI:** 10.3389/fonc.2024.1425822

**Published:** 2024-08-07

**Authors:** Gaoyang Cao, Xinjie Zhang, Songtao Wu, Wei Zhou

**Affiliations:** ^1^ Department of Colorectal Surgery, Sir Run Run Shaw Hospital, School of Medicine, Zhejiang University, Hangzhou, China; ^2^ School of Medicine, Zhejiang University, Hangzhou, China

**Keywords:** anastomotic stricture, occult, rectal cancer surgery, anal dilatation, diagnostic criteria

## Abstract

**Background:**

Anastomotic stricture (AS) is a common complication following rectal cancer surgery with anastomosis, but its diagnosis and management pose significant challenges due to the lack of standardized diagnostic criteria. We present a case highlighting the complexities encountered in diagnosing and managing occult AS post-rectal cancer surgery.

**Case presentation:**

A 51-year-old male patient presented with symptoms suggestive of AS following robot-assisted laparoscopic low anterior resection for rectal adenocarcinoma. Despite conventional evaluations, including colonoscopy, digital rectal examination, and radiography, AS was not identified. Following prolonged and ineffective treatment for suspected conditions such as low anterior resection syndrome (LARS), the patient underwent anal dilatation, resulting in significant symptom improvement.

**Conclusions:**

This case underscores the challenges associated with diagnosing and managing occult AS following rectal cancer surgery. The absence of standardized diagnostic criteria and reliance on conventional modalities may lead to underdiagnosis and inadequate treatment. A comprehensive diagnostic approach considering intestinal diameter, elasticity, and symptoms related to difficult defecation may enhance diagnostic accuracy. Further research is needed to refine the diagnostic and therapeutic strategies for occult AS.

## Background

Anastomotic stricture (AS) is a common complication following rectal cancer surgery with anastomosis. With the advancement of minimally invasive surgical techniques, chemoradiotherapy, and immunotherapy, an increasing number of patients with rectal cancer are opting for a sphincter-preserving surgery. Consequently, complications related to anastomosis are also on the rise, including AS. Its incidence ranges from 2% to 30% (1, 2). This wide range is mainly due to the lack of standardized definition for AS. While most studies define AS as difficulty in the passage of an endoscope through the anastomosis, the diameter of the endoscope used varied widely, ranging from 11 to 20 mm (3, 4). Some studies also consider AS as a narrowing less than one finger’s width upon digital examination (5, 6). Inconsistencies in finger width among clinicians further contribute to diagnostic differences in AS. Here we present a case of rectal anastomotic stricture where the index finger and colonoscopy passed readily. This case involved a middle-aged male patient experiencing symptoms resembling low anterior resection syndrome (LARS), including increased bowel movements and difficult and painful defecation. Despite consultations with renowned physicians nationwide, he was diagnosed with LARS, irritable bowel syndrome, or intestinal functional disorders and underwent over 2 years of prolonged and painful treatment, with no improvement in symptoms. Finally, after 1 month of anal dilatation treatment at our facility, his symptoms significantly improved.

## Case presentation

**Figure 1:**
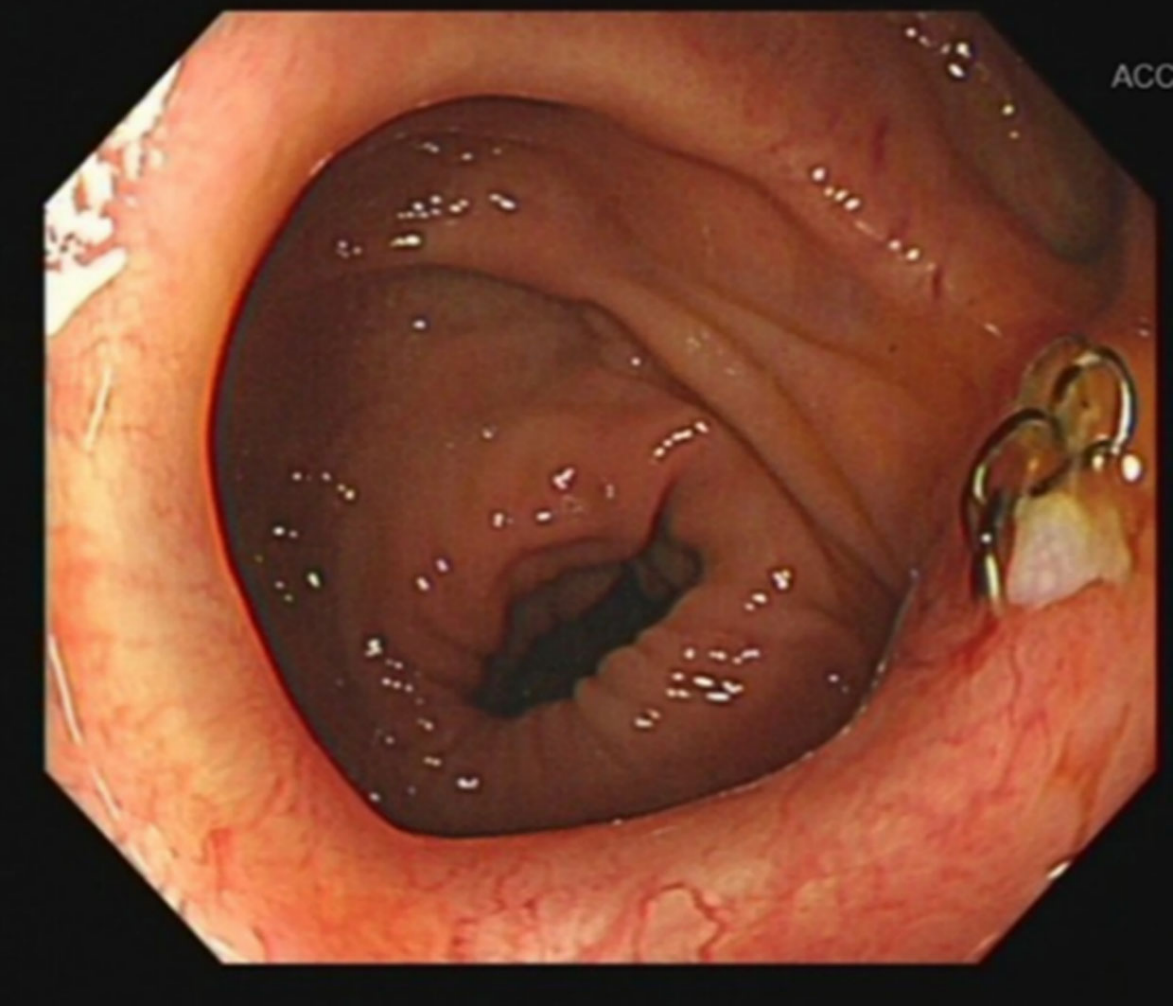
Colonoscopy showing anastomotic metal nails with no evidence of anastomotic stenosis.

A 51-year-old man was diagnosed with rectal adenocarcinoma staged as cT3N2M0 over 3 years ago. Due to concerns regarding potential side effects on genitourinary and bowel function, he underwent three cycles of preoperative neoadjuvant chemotherapy using the FOLFOXIRI regimen, omitting radiotherapy. This regimen consists of a combination of fluorouracil, leucovorin, oxaliplatin, and irinotecan. Upon reassessment, his tumor showed shrinkage compared to previous evaluations. Consequently, the patient underwent robot-assisted laparoscopic low anterior resection with protective ileostomy on March 22, 2021. The left colonic artery was preserved during the operation to ensure better blood supply to the anastomosis. Postoperatively, the pathology indicated moderately differentiated adenocarcinoma, staged as pyT2N0M0. His recovery was uneventful, and he received six cycles of Capeox chemotherapy, which consisted of capecitabine and oxaliplatin. Prior to ileostomy reversal, the patient underwent a comprehensive examination including colonoscopy and CT scans, which showed no stricture at the rectal anastomosis and no evidence of recurrence or metastasis. The ileostomy was reversed at 153 days. Although the ileostomy reversal proceeded smoothly, he experienced increased stool frequency, approximately seven to eight times per day. Over the following 2 years, his bowel movements gradually decreased to three to six times per day, but he experienced prolonged and laborious defecation, with each stool passage taking more than 30 min. Colonoscopy and abdominal CT scan did not reveal anastomotic stenosis (with a diameter exceeding 1.5 cm), as evidenced by the smooth passage of a colonoscope at the anastomosis site (**Figure 1**). Upon digital rectal examination (DRE), the anastomotic ring could be felt at a distance of 3 to 4 cm from the anus, and there was no hindrance when passing the index finger through the anastomotic site. These symptoms caused significant distress to the patient, nearly rendering him unable to work. Despite visits to multiple medical centers, experts found no evidence of anastomotic stenosis and diagnosed him with LARS and functional bowel disease. He underwent treatment with laxatives, probiotics, biofeedback, and pelvic floor rehabilitation exercises, but his symptoms showed no improvement.

**Figure 2:**
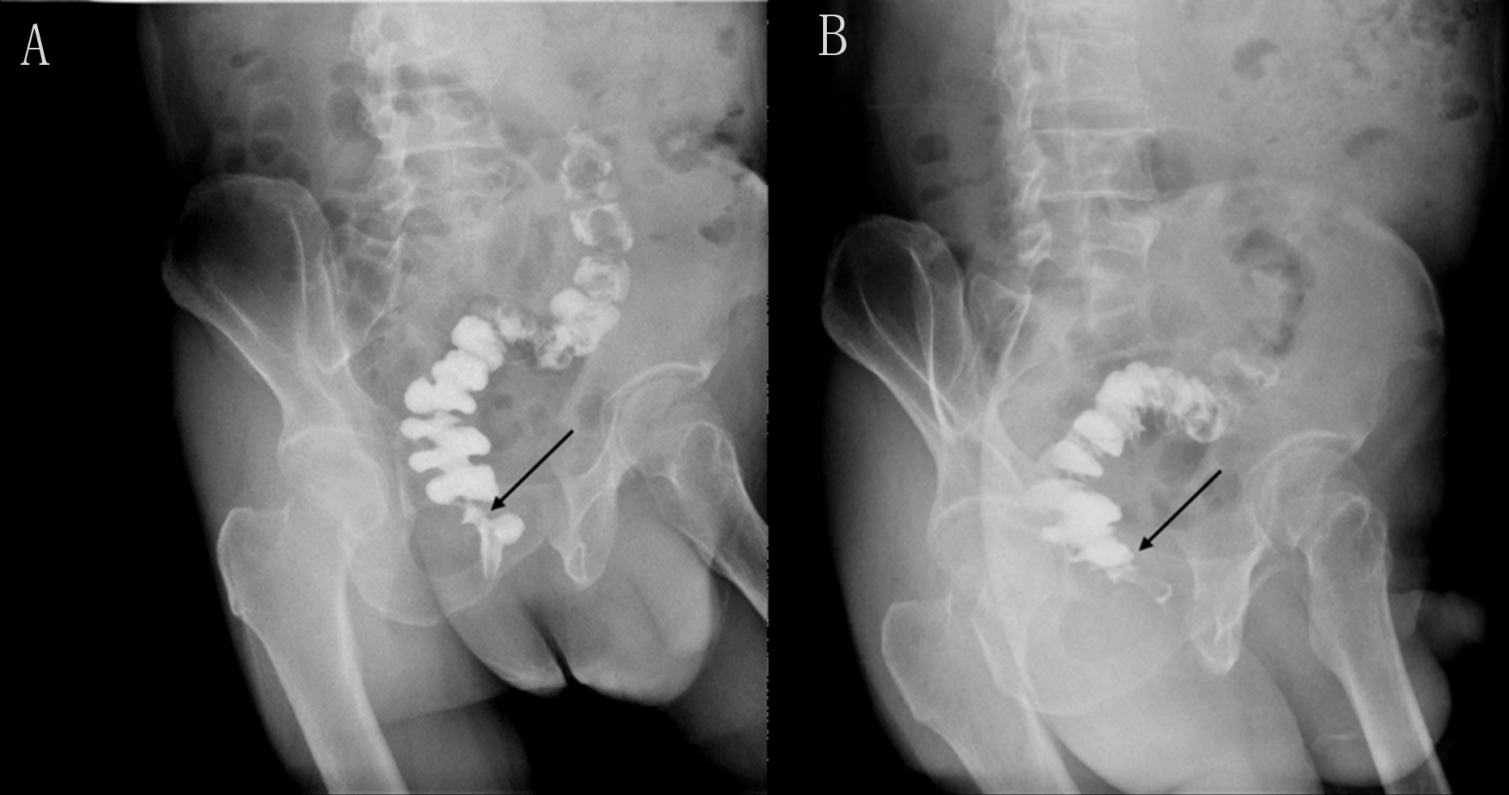
Rectal iodine contrast examination revealed no rectal anastomotic stricture. **(A)** Supine position. **(B)** Left lateral position. The arrow indicates the location of the anastomosis, which is wider than 1.2 cm.

**Figure 3:**
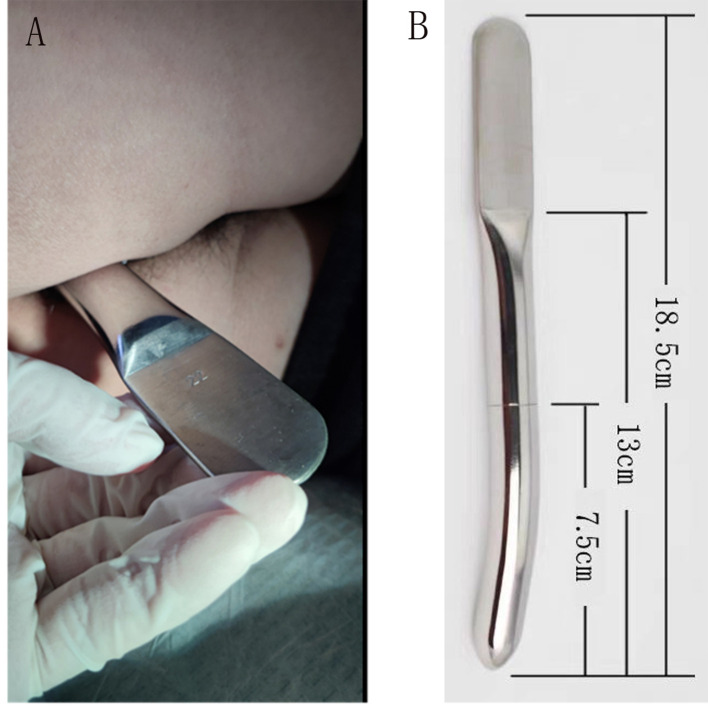
**(A)** Anal dilation using 22-mm-diameter Hegar’s dilator. **(B)** Hegar’s dilator.

Seeking further treatment, he approached our team. Pelvic floor electromyography revealed mild spasm of the puborectal muscle, with no significant abnormalities in the static tension or contraction strength of the anal sphincter muscles. This indicated that pelvic floor muscle dysfunction was not the primary cause of his difficulty in defecation. Meanwhile, rectal radiography also showed no evidence of anastomotic stricture (**Figure 2**). Upon digital rectal examination, it was found that the index finger passed readily, but the anastomotic ring was noted to be inelastic. Based on these findings, we hypothesized that although the anastomosis was large enough by traditional AS standards, it may have failed to functionally relax and expand during defecation, thus contributing to the difficulty in bowel movements. Therefore, we recommended instrumental anal dilatation due to its wide range of diameter and ease of operation. Hegar’s dilatation was initiated at 1.5 cm and gradually increased to 2.2 cm (**Figure 3**) until the patient felt discomfort. On the first day post-treatment, his symptoms significantly improved. However, recurrence occurred 2 days later. Therefore, the patient was advised to undergo Hegar’s dilatation every alternate day for 1 week, twice a week for another week, and once a week for the following 2 weeks. Subsequently, the patient experienced smoother voluntary defecation without the need for laxatives in the subsequent 6 months. We continue to monitor the patient closely at our outpatient department. The timeline of patient care and key interventions is provided in **Supplementary Table S1**.

## Discussion

The patient’s journey began with a diagnosis of rectal adenocarcinoma, leading to robot-assisted laparoscopic low anterior resection. Following surgery, he experienced increased stool frequency and prolonged defecation, severely impacting his quality of life. Despite consulting multiple specialists and receiving various treatments, his symptoms persisted. Identifying an inelastic anastomotic ring led to the recommendation of anal dilatation, which significantly improved his symptoms. The patient expressed relief and gratitude for the eventual resolution, enabling him to resume daily activities and work. His experience underscores the need for comprehensive diagnostic approaches and individualized treatment plans in managing occult AS.

The presented case highlights several challenges encountered in the diagnosis and management of AS following rectal cancer surgery. While AS is typically defined as difficulty in passing an endoscope or index finger through the anastomosis, the conventional criteria for diagnosing AS may fail to capture subtle abnormalities in anastomotic function, as observed in this case. The failure of the anastomosis to effectively relax and expand during defecation, despite meeting the traditional size criteria, suggests a functional rather than structural etiology. Currently, there is limited literature that considers dynamic factors, such as anastomotic compliance and contractility, in the evaluation of AS. The AS definition described by Jinchun et al. (6), based on an analysis of 473 post-rectal cancer surgery patients, categorized AS into five degrees. This classification explicitly uses intestinal diameter and elasticity as the main assessment criteria for AS. However, this approach may lead to overdiagnosis, as evidenced by AS diagnosis rates of 88% and 86% at 3 and 6 months postoperatively. This high rate may be attributed to the exclusion of symptoms, such as excessive straining and sensation of incomplete evacuation or blockage, from the diagnostic criteria. In this case, despite experiencing symptoms such as sensation of incomplete evacuation and excessive straining, the patient’s increased bowel movements masked the diagnosis of AS. Therefore, we suggest that the diagnosis of AS should incorporate not only intestinal diameter and elasticity but also symptoms related to difficult defecation. This comprehensive approach to diagnosis may improve the accuracy of AS diagnosis and facilitate appropriate management strategies.

Though AS diagnosis is generally straightforward, it may occasionally be confounded by symptoms of other condition like LARS, potentially delaying treatment. While LARS shares symptoms such as emptying difficulties and painful stools with AS, it primarily manifests as increased stool frequency and incontinence, contrasting with AS symptoms (7). However, LARS and AS can coexist or alternate. Fecal continence relies on multiple factors, including anatomy, rectal compliance, anorectal sensation, pelvic floor muscle strength, stool consistency, mobility, and psychological factors (8). The delicate balance between constipation, characteristic of AS, and incontinence, common in LARS, may be disrupted by pelvic floor dysfunction resulting from disturbed muscle tone or overactive pelvic floor muscle (9), which are frequently observed post-rectal surgery. This dysfunction may contribute to the intermittent occurrence of both difficult defecation and fecal incontinence. In this case, the patient exhibited symptoms consistent with LARS, including increased bowel movements and painful defecation, suggestive of AS. However, despite extensive consultations and investigations, including colonoscopy and computed tomography (CT) scans, AS was not identified. The index finger passed readily on DRE, and radiographic imaging showed no evidence of anastomotic stenosis. This discrepancy between the clinical symptoms and the diagnostic findings underscores the limitations of current diagnostic modalities in detecting occult AS. Therefore, during the diagnosis and treatment of AS, it is important to consider the possibility of concurrent LARS. In this patient, the persistence of symptoms such as increased bowel movements and urgency after anal dilation suggests the presence of mild-to-moderate LARS alongside AS. However, particular caution is warranted during AS treatment, especially when choosing anal dilation, to prevent exacerbating LARS.

Successful symptom resolution post-anal dilation supports the functional nature of the observed obstruction, indicating instrumental intervention as a therapeutic option for occult AS. Treatment options for AS include mechanical dilation, endoscopic treatments, and surgical procedures (10). The choice of optimal treatment depends on the doctor’s judgment and the characteristics of AS. Mechanical dilation, including digital and Hegar’s dilatation, is the simplest and most commonly used method but may require multiple sessions for treatment goals to be achieved (11, 12). In this case, the patient underwent a total of eight sessions within 4 weeks to achieve satisfactory results, likely attributable to delayed treatment initiation 3 years after symptom onset. However, the optimal timing and frequency of dilatation warrant further investigation to minimize recurrence and optimize long-term outcomes. Hegar’s dilatation should be performed cautiously to avoid perforation, as reported in the literature (2). Endoscopic treatments like balloon dilatation and self-expansion stent are suitable for AS patients with poor mechanical dilation efficacy or when inaccessible by digital rectal examination (13) but carry the risks of complications such as perforation, hemorrhage, and leakage (14, 15). Transanal endoscopic microsurgery (TEM) (16) and transanal minimally invasive surgery (TAMIS) (17) have gradually been used for the surgical treatment of AS, offering superior surgical vision and precise incision, especially for mid- and upper rectal AS.

## Conclusions

This case underscores the challenges associated with diagnosing and managing occult anastomotic stricture (AS) following rectal cancer surgery. The absence of standardized diagnostic criteria and dependence on conventional modalities, such as colonoscopy and digital rectal examination, may contribute to underdiagnosis and inadequate treatment of AS. A comprehensive approach incorporating intestinal diameter, elasticity, and symptoms related to difficult defecation may offer a more effective diagnostic strategy for AS. Further research is warranted to elucidate the pathophysiology of occult AS and to refine diagnostic and therapeutic strategies accordingly.

## Data availability statement

The original contributions presented in the study are included in the article/**Supplementary Material**. Further inquiries can be directed to the corresponding author.

## Ethics statement

The studies involving humans were approved by Medical Ethical Committee of Sir Run Run Shaw Hospital. The studies were conducted in accordance with the local legislation and institutional requirements. Written informed consent for participation was not required from the participants or the participants’ legal guardians/next of kin in accordance with the national legislation and institutional requirements. Written informed consent was obtained from the individual(s) for the publication of any potentially identifiable images or data included in this article.

## Author contributions

GC: Writing – review & editing, Writing – original draft, Funding acquisition. XZ: Writing – review & editing, Writing – original draft, Funding acquisition. SW: Writing – original draft, Visualization, Formal analysis. WZ: Writing – review & editing, Supervision, Funding acquisition, Formal analysis, Conceptualization.
