# Supplementary material for: Resolution of occult anastomotic stricture with anal dilator: challenges with the conventional diagnostic criteria in low anterior rectal resection patient—a case report
Source: Front Oncol. 2024 Aug 7;14:1425822. doi: 10.3389/fonc.2024.1425822 (PMC11335538; doi:10.3389/fonc.2024.1425822)
Supplement: Supplementary file 1 [file Table_1.docx]

**Supplementary Table 1: Timeline of Patient Care and Key Interventions**

| **Date** | **Event/Intervention** | **Details** |
| --- | --- | --- |
| **March 22, 2021** | Surgery | Robot-assisted laparoscopic low anterior resection with protective ileostomy for rectal adenocarcinoma |
| **Post-Surgery** | Pathology Results | Moderately differentiated adenocarcinoma staged as pyT2N0M0 |
| **Post-Surgery** | Chemotherapy | Six cycles of Capeox (capecitabine and oxaliplatin) |
| **Two Years Post-Surgery** | Symptom Onset | Increased stool frequency (7-8 times/day), later reducing to 3-6 times/day; prolonged defecation (>30 minutes) |
| **Multiple Evaluations** | Diagnostics | Colonoscopy and CT scans show no anastomotic stenosis; diagnosed with LARS, IBS, and functional bowel disease |
| **After Two Years** | Further Evaluations | Consultations with multiple experts; no anastomotic stenosis found |
| **Recent Evaluation** | Pelvic Floor Electromyography | Mild puborectal muscle spasm; no significant anal sphincter abnormalities |
| **Recent Evaluation** | Digital Rectal Examination (DRE) | Inelastic anastomotic ring felt; hypothesis of functional relaxation failure |
| **Recent Treatment** | Anal Dilatation (Hegar’s Dilator) | Initial dilatation at 1.5 cm, increased to 2.2 cm; significant symptom improvement but recurrence observed |
| **Follow-Up Treatment** | Continued Dilatation | Dilatation every alternate day for one week, twice a week for another week, and once a week for two weeks |
| **Outcome** | Post-Treatment | Smoother voluntary defecation; no need for laxatives; monitored regularly |
